# The effect of drying methods on the energy consumption, bioactive potential and colour of dried leaves of Pink Rock Rose (*Cistus creticus*)

**DOI:** 10.1007/s13197-019-03656-2

**Published:** 2019-03-22

**Authors:** Agnieszka Ewa Stępień, Józef Gorzelany, Natalia Matłok, Krzysztof Lech, Adam Figiel

**Affiliations:** 1 Centre for Innovative Research in Medical and Natural Sciences, Medical Faculty, University of Rzeszow, Warzywna 1A, 35-310 Rzeszów, Poland; 2 Department of Dietetics, Institute of Nursing and Health Sciences, Medical Faculty, University of Rzeszow, Al. mjr.W.Kopisto 2 a, 35-310 Rzeszów, Poland; 3 Department of Food and Agriculture Production Engineering, Faculty of Biology and Agriculture, University of Rzeszow, St. Zelwerowicza 4, 35-601 Rzeszów, Poland; 4 Institute of Agricultural Engineering, Wroclaw University of Environmental and Life Sciences, Chelmonskiego 37a, 51-630 Wroclaw, Poland

**Keywords:** Pink Rock Rose (*Cistus creticus*), Antioxidant activity, Total polyphenolic content, Drying

## Abstract

This study aimed to investigate the changes in the total polyphenolic content and antioxidant properties after subjecting Pink Rock Rose (*Cistus creticus*) leaves to three different drying procedures, including convection drying (CD) at 40, 50 and 60 °C; vacuum-microwave drying (VMD) at 240 W microwave power; and combined drying consisting of convective pre-drying at 50 °C followed by vacuum-microwave finish drying at 240 W microwave power (CPD-VMFD). The total polyphenolic content and antioxidant properties (DPPH, ABTS) of leaves subjected to these three drying methods were spectrophotometrically determined. The results show that convection drying at 40 °C and vacuum-microwave drying yielded dried leaves with the highest bioactive potential in terms of the total polyphenol content and antioxidant activity, with the highest and lowest values of final specific energy consumption, respectively. The lowest bioactive potential was found in a product dried at 60 °C, which can be attributed to the possible degradation or changes in polyphenol structures under high temperatures. During the combined treatment (CPD-VMFD), most of the moisture was efficiently removed from the raw material by CPD, whereas the time of drying was significantly reduced by the application of VMFD. Combined drying CPD-VMFD is most suitable for industrial applications as it produces dried leaves with a bioactive potential that is only slightly lower than that achieved with VMD while providing a high-throughput capacity relative to operating costs.

## Introduction

The *Cistus* species is an herb particularly valuable to human health representing the *Cistaceae* family. It is a perennial shrub native to the Mediterranean, Europe and western Asia. The valuable therapeutic properties of the *Cistus* species include anti-inflammatory, antibacterial, antifungal, antiviral, anti-allergic, antioxidant and antitumor effects; it also improves immunity and is known for its analgesic effects (Stępień 2017).

Studies indicate that the Pink Rock Rose (also called Cistus gray, *Cistus creticus* L., *son. Cistus villosus*, *Cistus polymorphus*, and *Cistus incanus creticus*) exhibits antimicrobial properties, and its oils and extracts show valuable antioxidant properties (Stępień et al. 2018). Raw herbs, like other plant materials with high moisture content, are susceptible to deterioration of their quality or even spoilage due to microbial processes, and therefore they require adequate preservation. Drying is a method that prevents these undesirable processes. However, during drying raw materials undergo changes which most often lead to a deterioration of the quality and properties of processed herbs.

The drying of herbs inhibits the growth of microorganisms as well as some biochemical transformations, but it can also negatively impact the final quality of the product. Changes in appearance and aroma often indicate or are concomitant with the partial or complete loss of bioactive compounds that determine the antioxidant properties of plant products (Hossain et al. 2010). For example, the content of phenolic compounds contributing to antioxidant plant properties is reduced as the result of the oxidation processes and high temperature used during drying (Oszmiański et al. 2007).

The most common drying techniques used for the preservation of herbs include convection drying and vacuum-microwave drying in a vacuum environment (Lin et al. 2013) as well as convection-microwave drying, which is a combination of these two methods applied either simultaneously or in succession where convection pre-drying is followed by vacuum-microwave finish drying (Figiel 2010).

In case of herbal plants, it is critical to determine the optimal conditions of the drying process so that any adverse changes in the chemical composition of the raw materials are minimized to preserve their most valuable bioactive properties. For industrial applications, the drying process should assure the best possible quality of the final product in terms of antioxidant properties, the content of polyphenolic compounds, and the colour of leaves preserved at possibly low costs associated with the energy consumption.

The purpose of this work was to determine the impact of parameters of the drying process on the energy consumption and antioxidant properties (DPPH, ABTS), total content of polyphenolic compounds, and the colour of the dried product obtained from Pink Rock Rose leaves. The drying methods used in this work included convection drying (CD), vacuum-microwave drying (VMD) and a combination of both, i.e., convection pre-drying followed by vacuum-microwave finish drying (CPD-VMFD).

## Materials and methods

### Plant materials

*Cistus creticus* plants were cultivated under ecological conditions in a garden tunnel located in a nursery farm in the city of Rzeszów (Poland). *Cistus creticus* leaves were harvested in the second year of cultivation. The dry matter content in the fresh material was 19.56%, corresponding to the water content of 80.44%. The plant leaves were then processed in the laboratory of the Wroclaw University of Environmental and Life Sciences (WUELS) using different drying methods.

### Reagents

DPPH (2,2-diphenyl-1-picrylhydrazyl free radical) (> 98%), ABTS (2,2′-azino-bis(3-ethylbenzothiazoline-6-sulphonic acid), Trolox^®^, and gallic acid were purchased from Sigma-Aldrich (Steinheim, Germany), and potassium persulphate, phosphate buffered saline solution pH = 7.4, Folin–Ciocalteu’s phenol reagent (acids 1.9–2.0 mol/dm^−3^), ethanol 96%, and sodium carbonate were purchased from Chempur (Gliwice, Poland).

### Methods of drying

Samples of raw *C. creticus* leaves weighing 60 g ± 1 mg were dried using three methods: convection drying (CD), vacuum-microwave drying (VMD) and combined drying, involving convection pre-drying and vacuum-microwave finish drying (CPD-VMFD).

Convection drying (Figiel et al. 2010) was performed in a convection dryer designed and built at the Institute of Agricultural Engineering (WUELS, Poland). The temperature and air velocity were 40 °C, 50 °C and 60 °C and 0.8 m·s^−1^, respectively. To obtain an even distribution of experimental points while the dehydration process transpired with a decreasing drying rate, weight measurements were performed after 5, 10, 15, and 30 min and then every 60 min until a final moisture of 7% wb was reached considering the initial fresh moisture content of 77%. The weight of the drying samples was determined using a laboratory scale with an accuracy of ± 0.05 g.

The VMD process (Wojdyło et al. 2009) was performed in a Plazmatronika SM 200 dryer (Wroclaw, Poland) at the constant magnetron output power of 240 W. The samples were placed in a 6.8-L cylindrical organic glass container. The pressure in the container varied from 4 to 6 kPa. To avoid local over-heating of the plant material, the container rotated at a speed of 6 rpm and the samples were dried using an intermittent method of 4-min cycles. After each cycle the material was removed from the drying chamber for measurements of the temperature and mass. The temperature was measured using a Flir i50 infrared camera (FLIR Systems, Sweden) with an accuracy of ± 2 °C. The drying process was stopped when the sample mass measurement performed with an accuracy of ± 0.05 g showed a value corresponding to the assumed final moisture content of 7% wb.

The CPD-VMFD method (Calín-Sánchez et al. 2014) involved pre-drying of the samples for 3 h in a convection dryer at a temperature and airflow of 50 °C and 0.8 m s^−1^, respectively; subsequently, additional drying was performed in a vacuum-microwave dryer at a power of 240 W magnetrons in a rotating drying chamber until the sample mass measurement (0.05 g accuracy) showed a weight value corresponding to the assumed final moisture content of 7% wb. All drying tests were performed in triplicate.

### Modelling of drying kinetics

Modelling of the drying kinetics was performed using Table Curve 2D (Systat Software, San Jose, California, USA), which enabled fitting of the modified Page’s model (1) to experimental points. Page’s model is often used to predict the decrease in moisture ratio (*MR*) versus the time of drying (*t*) taking into account the highest values of the coefficient of determination R^2^ and the lowest values of the root mean square error (RMSE) when compared with the fitting results using other drying models.

1 $$ MR = A \cdot e^{{ - k \cdot t^{n} }} $$

where *A*—coefficient corresponding to the initial value of *MR*, *k*—drying constant, *n*—exponent.

The moisture ratio (*MR*) was expressed as the ratio of the current moisture content to the initial moisture content (2), as measured by a gravimetric method (Alibas 2006).

2 $$ MR = \frac{M}{{M_{0} }} $$

where *M* is the current moisture content. *M*_0_ is the initial moisture content.

The value of *M*_0_ was determined gravimetrically taking into account the initial mass of sample and dry matter content after drying for 24 h at 60 °C in the SPT-200 vacuum dryer (ZEAMiL Horyzont, Krakow, Poland). The values of *M* were predicted according to the value of *M*_0_ and the current mass of the dried samples. The value of the final moisture content was confirmed gravimetrically. The mass of the samples was determined using an analytical balance XA 60/220X with an accuracy of 0.001 g.

### Energy consumption

The energy *E*_*C*_ consumed during convective drying (kJ) was calculated according to Equation (3):

3 $$ E_{C} = \left( {\frac{{N_{f} }}{6} + N_{h} } \right) \times t $$

where *N*_*f*_ (kW) is the power consumption by a fan blowing air into the six pipes equipped with electric heaters of power consumption *N*_*h*_ (kW), and *t* is the time of drying (s).

The energy *E*_*VM*_ consumed during VM drying (kJ) was calculated according to Equation (4):

4 $$ E_{VM} = \left( {\frac{{N_{M} }}{{\eta_{M} }} + N_{V} + N_{e} } \right) \times t $$

where *N*_*M*_ and *η*_*M*_ are the output power (kW) and efficiency of magnetrons, respectively; (*N*_*V*_) is the power consumption (kW) by the vacuum pump, and (*N*_*e*_) is the power consumption (kW) by the electric engine rotating the container.

The specific energy consumption (*E′*) was calculated as the ratio of energy consumed during drying to the mass of water removed from the fresh sample during this process (Calín-Sánchez et al. 2014).

### Plant extract

The weight of each sample was calculated according to the dry matter content for both the dried product and the raw material. An equivalent of 0.5 g of dry matter of each sample was then extracted by stirring with 10 ml of 70% aqueous ethanol for 30 min at room temperature as described by Mahmoudi et al. (2016). All extractions were performed in triplicate.

### Total polyphenolic content

The total polyphenolic content was determined using the Folin-Ciocalteu reagent method as described by Prior et al. (2005) with some modifications. A 125 μl aliquot of the diluted extract was added to 2.375 ml deionized water and 125 μl Folin-Ciocalteu reagent (diluted 1:1 with water, acids 0.8–1.0 mol/dm^−3^). After shaking, the mixture was incubated for 3 min at room temperature. Then, 250 μl of 7% Na_2_CO_3_ solution was added; after mixing, the solution was stored for 30 min at RT in dark. The absorbance at 760 nm was measured in triplicate using a Thermo Scientific Evolution spectrometer. The results were corrected for the dilution of gallic acid [mg/ml] as a standard.

### Antioxidant activities

#### DPPH method

The DPPH free radical scavenging method described by Yen and Chen (1995) was applied with modifications. Then, 1.2 ml of ethanol solution of DPPH (3 mM) was added to 0.3 ml of the polyphenol solution extract. The mixture was shaken and stored in dark for 10 min, and then the absorbance at 517 nm was measured using a Thermo Scientific Evolution spectrophotometer in three technical replicates. The results were corrected for the dilution of Trolox [mg/100 ml] as a standard.

#### ABTS method

The free radical scavenging activity was determined by ABTS according to Re et al. (1999). The absorbance was measured at 734 nm with a Thermo Scientific Evolution spectrometer in triplicate. The results were corrected for the dilution of Trolox [mg/100 ml] as a standard.

### Colour change

The *C. creticus* leaf colour was measured in a CIE L*a*b* system reflected in light using a Colour Ques spectrophotometer (HunterLab, USA) by introducing samples into a cuvette with an optical path length of 10 mm. The spectra were developed in the Easy Mach QC program for a 10º viewing angle and the D65 light source. The following colour parameters were determined: L*—brightness, a*—red and green (redness and greenness), and b*—yellow and blue (yellowness and blueness). In addition, the colour differences (ΔE) of the leaves subjected to drying were calculated assuming fresh material as the colour reference. The mean value of three replicates was taken as the result of the determination.

### Statistical analysis

A one-way analysis of variance (ANOVA) and multiple pairwise Tukey test was applied in Statistica 13. Statistical analyses of the colour change results were performed using Student’s *t* test in Statistica 13.1.

## Results and discussion

### The kinetics of drying

**Fig. 1:**
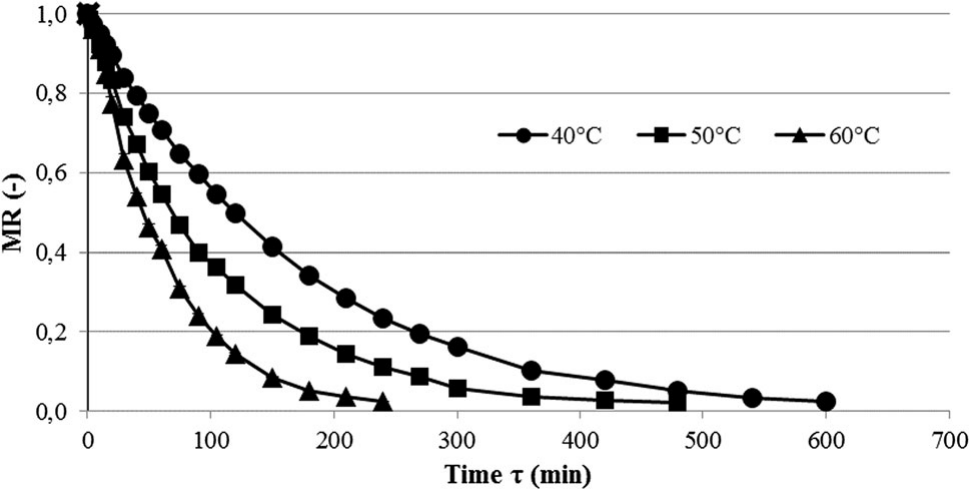
Water content decrease in *Cistus creticus* leaves during convection drying (CD) at air temperatures of 40, 50 and 60 °C

The drying kinetics of *C. creticus* leaves using CD at defined drying temperatures is shown in Fig. 1, and the parameters of the Page model fitted to the empirical points are summarized in Table 1. The application of temperatures of 40 °C, 50 °C and 60 °C allowed dried products to be obtained with the final water contents of 7.8, 7.3 and 7.5%, respectively, following 600 min, 480 min, and 240 min of drying (Table 1). A change in the temperature from 40 °C to 60 °C resulted in a nearly twofold increase in the drying constant k from 0.005 to 0.009 with a slight increase in the exponent n from 1.03 to 1.12, which is related to an increase in the drying rate and consequently a possible decrease in the drying time. Notably, an increase in the drying temperature by 10 °C from 40 °C to 50 °C and from 50 °C to 60 °C resulted in a reduction of the drying time by 20% and 50%, respectively. This disproportionate nature of the beneficial effect of the air temperature in reducing the drying time is due to an increase in the diffusion coefficient of water which depends on the temperature of the material (Bialobrzewski and Markowski 2004). Convection drying is a relatively time-consuming method compared with microwave-assisted methods (Figiel and Michalska 2017).

**Table 1:** The Page’s model parameters, drying time and final water content in dried *Cistus creticus* in convection drying (40 °C-CD 40 °C, 50 °C-CD 50 °C, 60 °C-CD60 °C), vacuum-microwave (VMD 240 W) and vacuum-microwave drying after convection pre-drying (CPD 50 °C-VMFD 240 W)

Drying conditions	Constants			Statistics		Drying time (min)		*Mc*_wb_ (%)
	*A*	*k*	*n*	RMSE^*‡*^	R^2^	*CD*	VMD	
CD 40 °C	1	0.005	1.03	0.003	0.9999	600	–	7.8
CD 50 °C	1	0.011	0.97	0.01	0.9989	480	–	7.3
CD 60 °C	1	0.009	1.12	0.015	0.9981	240	–	7.5
VMD 240 W	1	0.01	1.65	0.028	0.9932	–	48	6.4
CPD 50 °C-VMFD 240 W	0.244	0.601	0.467	0.006	0.9914	150	24	7.4

**Fig. 2:**
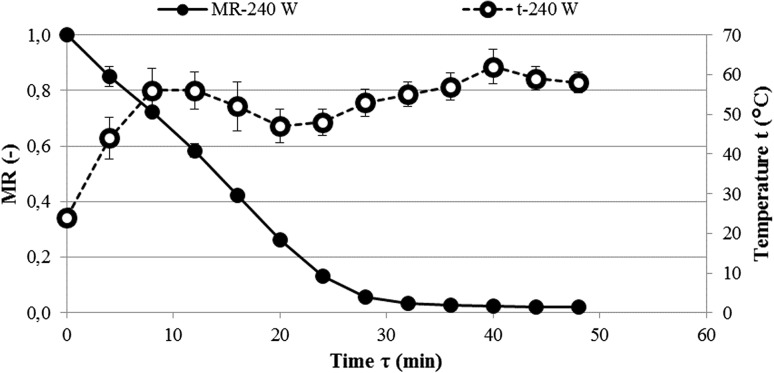
Water content decrease in *Cistus creticus* leaves during vacuum-microwave drying (VMD)

**Fig. 3:**
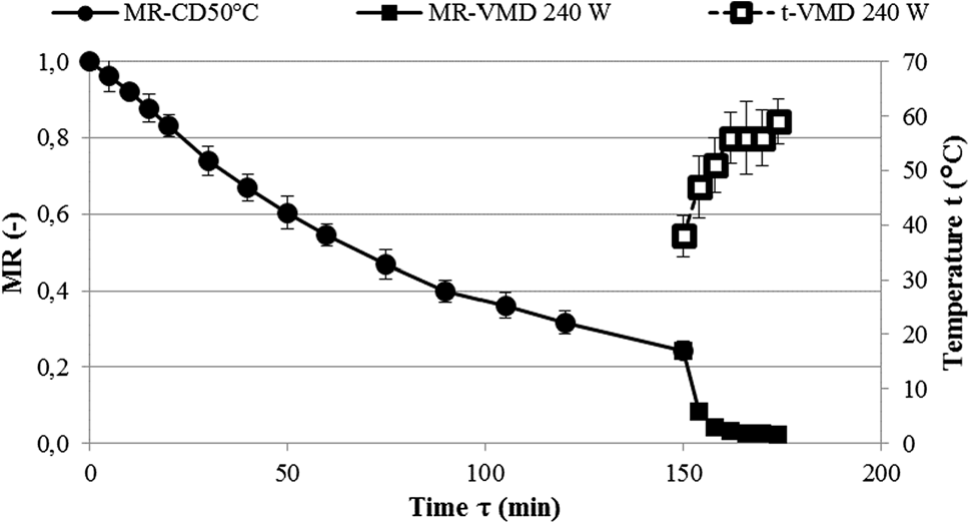
Water content decrease in *Cistus creticus* leaves during vacuum-microwave drying after convection pre-drying (CPD) at 40 °C followed by VMFD

This observation was also confirmed by the results of the present study. For example, in the VMD process, at the power of 240 W magnetrons, the water content of *C. creticus* leaves reached a final value of 6.4% just after 48 min (Fig. 2). Such a short drying time is related to the high drying rate, which is evidenced by the high drying constant *k* of 0.01 at the exponent n of 1.65 (Table 1). As a result of the high drying rate, the actual final moisture content of the sample was lower than the assumed theoretical value of 7%. The high rate of drying associated with the internal microwave heating at reduced external pressure is because the diffusion of water in accordance with Fick’s law is supported by the pressure transport mechanism of the Darcy type (Lech et al. 2015). Both the water diffusion and the pressure transport mechanism are conducive to the temperature of the dried material, measured using an infrared camera. The initial rise in leaf temperature to 56 °C followed by fluctuations to reach a maximum of 62 °C just before the end of the drying process characterized by a drop to 58 °C (Fig. 2) is due to the balance of the energy generated by the water molecule dipoles inside the microwaved material and the necessary energy for the evaporation of water from the surface of the material (Figiel 2010). This pattern of drying by vacuum-microwaves is characteristic of plant materials (Calín-Sánchez et al. 2014). During combined drying (Fig. 3), CPD lasting 150 min resulted in a decrease in the water content of the sample to 0.844 kg·kg^−1^ dm, corresponding to the moisture ratio MR = 0.244 associated with the A value (Table 1) in the modified Page model of VMFD lasting 28 min to reach a final water content of 7.4%. The nature of the changes in the temperature of the material during VMFD was similar to that of VMD, with its maximum value being slightly lower and reaching 59 °C. When analysing the drying kinetics in terms of the temperature and duration of its effects, however, the use of high temperatures leads to changes in the colour, the contents of active compounds, and the texture of products of plant origin (Śledź et al. 2013).

### Energy consumption

**Fig. 4:**
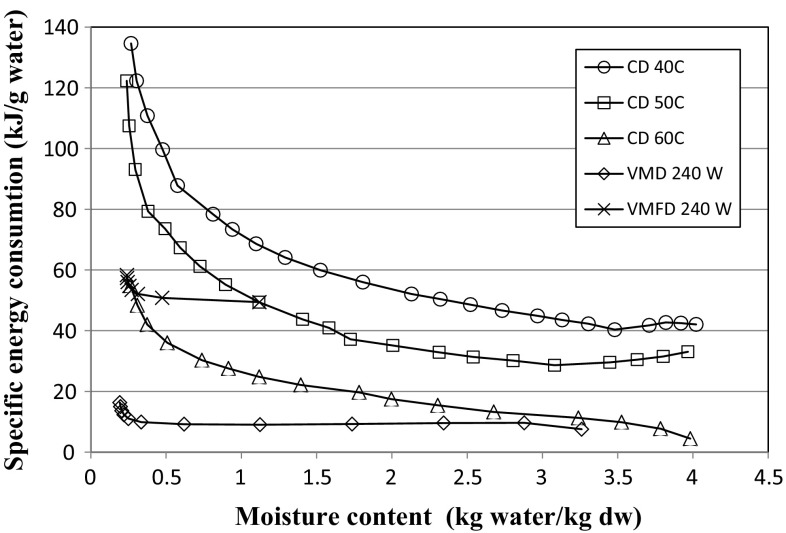
Specific energy consumption during CD, VMD and the combined drying (CPD) at 40 °C followed by VMFD of *Cistus creticus* leaves

The specific energy consumption E*′* for drying Pink Rock Rose leaves using CD, VMD and the combination of CPD-VMFD is shown in Fig. 4. The values of E*′* represent the amount of energy per gram of water removed from the fresh material during the drying process. The response of the E*′* profile shows an increase in the energy demand at the last stages of the drying process, and this behaviour is typical for plant materials with osmotic cellular structure (Sarsavadia 2007). This profile also indicates that as the drying process occurs, increasingly more energy is required to remove the same amount of water. However, at the beginning of the CD process, the shape of the E*′* curve exhibited a decreasing or increasing trend depending on the temperature of the hot air. This can be related to the shape of drying curves, starting with a more or less pronounced warming up period of the fresh material.

The highest value of E*′* attained at the end of drying amounted to 134.61 kJ g^−1^ and was found for CD at the lowest drying temperature of 40 °C. The increase in the drying temperature decreased the final value of E*′* to 54.81 kJ g^−1^ at the highest drying temperature of 60 °C. The lowest final value of E*′* amounted to 16.33 kJ g^−1^ and was achieved by VMD, which was performed at 240 W. On the other hand, the combined CPD-VMFD drying resulted in the final value of E*′* as 58.33 kJ g^−1^, which was comparable with the final value of E*′* obtained by CD at 60 °C. This order of final E*′* values is typical for plant materials subjected to different drying protocols (Calín-Sánchez et al. 2014).

### Antioxidant activities

During convective drying at 40 °C, 50 °C and 60 °C, the total polyphenolic content decreased by 81.78, 83.28 and 92.2%, respectively, compared with the raw Pink Rock Rose leaves. There was also a change in the antioxidant capacity in the DPPH by 84.70, 84.77, and 86.23%, respectively, and for the ABTS method the change was 79.33, 80.77 and 85.12%, respectively, in the case of the fixed drying temperatures: 40 °C, 50 °C, and 60 °C (Table 2). The findings show a notable decrease in the bioactive properties of Rock Rose leaves as a result of convection drying at 60 °C. It has been reported earlier that the contact of the material subjected to drying with the hot air causes degradation of important flavours and bioactive substances (Kramkowski 2001). In the course of drying, a relatively long oxidation process occurs at elevated temperatures, leading to significant losses of polyphenols, sometimes even up to 50% (Horubała 1999). For example, the convection drying of fresh blueberries at 70 °C resulted in a 39% loss of polyphenols and in a reduction of antioxidant capacity by 41% (Scibisz and Mitek 2006). In our study, during the vacuum-microwave drying of fresh Pink Rock Rose leaves, the content of polyphenols was reduced by 81.47%, and the bioactive potential was reduced by 84.65% and 80.8% as determined using the DPPH and ABTS methods, respectively (Table 2).

**Table 2:** Bioactive potential (DPPH test, ABTS test), and total polyphenolic content in dried *Cistus creticus* leaves obtained as a result of convection (40 °C, 50 °C, 60 °C), vacuum-microwave (VMD 240 W), and vacuum-microwave drying after convection pre-drying (CPD 50 °C-VMFD 240 W)

Drying conditions	DPPH^1^ (Trolox equivalent mg/100 ml) ± SD	ABTS^1^ (Trolox equivalent mg/100 ml) ± SD	Total phenolic content^1^ (gallic acid equivalent mg/ml) ± SD
Fresh	195.76 ± 0.01^a^	1916.59 ± 0.10^a^	76.2 ± 0.02^a^
CD 40 °C	29.96 ± 0.61^b^ (84.7%)	396.25 ± 2.69^b^ (79.33%)	14.02 ± 0.70^b^ (81.78%)
CD 50 °C	29.81 ± 0.37^b^ (84.77%)	368.68 ± 1.20^c^ (80.77%)	12.71 ± 0.01^c^ (83.28%)
CD 60 °C	26.95 ± 0.21^b*^ (86.23%)	284.87 ± 0.59^d^ (85.12%)	7.46 ± 0.39^d^ (92.2%)
VMD 240 W	30.04 ± 0.64^b*^ (84.65%)	366.47 ± 1.37^d*^ (80.88%)	14.09 ± 0.07^b^ (81.47%)
CPD 50 °C-VMFD 240 W	29.95 ± 0.46^b^ (84.7%)	373.48 ± 2.12^d*^ (80.52%)	13.96 ± 2.42^b^ (81.64%)

Drying value changes in the dried material are expressed as a percentage of the relevant value determined in the raw material

±SD i n = 3

^1^Statistical (*P* < 0.01) differences between the methods, assessed by ANOVA and post hoc multiple pairwise Tukey test, are indicated by different letters. *Difference at *P* < 0.05

The use of microwaves under vacuum conditions also reduced the bioactive potential of Rock Rose leaves but to a lesser extent than the convection method according to the DPPH values and polyphenol content (Table 2). This suggests that the acceleration of the microwaves-assisted drying process leads to a reduction of negative effects caused by biochemical changes due to the shorter drying time and consequently shorter exposure of the material to oxygen at the temperature of the material, which after heating up fluctuates between 50 and 62 °C (Kramkowski 2001). According to a study in which the effects of a convection-microwave drying method on the leaves of parsley were examined, the preservation of chlorophyll and polyphenols was achieved mainly by the shortest possible duration of drying and by optimizing the drying temperature (Śledź et al. 2013).

During microwave drying, the material is heated rapidly as the electromagnetic waves pass through the interior of the food (Meda et al. 2008). The advantages of microwave drying include, but are not limited to, shorter drying times, smaller changes in the colour and fragrance, and, most importantly, smaller loss of active ingredients. As already shown by others, microwave drying leads to a lower level of chlorophyll degradation in herbs than convection drying. This suggests that a final product of higher quality can be obtained using rapid and effective dehydration compared with the products obtained using other methods (Meda et al. 2008). However, despite the low energy consumption in the experiments reported here, VMD requires electrical energy, which is relatively expensive. Moreover, under industrial conditions, capital expenditures are associated with the construction of large vacuum installations equipped with microwave generators powered by relatively expensive electricity. These disadvantages of vacuum-microwave drying can be minimized by using convection pre-drying. As the result of the removal of significant amounts of water during the initial convective pre-drying phase, the mass and volume of the material to be dried by vacuum-microwaves is significantly reduced (Hu et al. 2006), which enables the reduction of both capital expenditures and operating costs. Pre-drying of the raw material using the convection method not only reduces the total cost of drying but also contributes to the higher quality of certain dried vegetables and fruits, for example, dried tomatoes (Durance and Wang 2002) and strawberries (Böhm et al. 2006). The results of the present study also show that the values obtained for CPD-VMFD and VMD were similar at baseline and showed no significant differences (Table 2). Similar results were reported by Figiel et al. (2010), who showed that the quality features of oregano samples dehydrated by CPD-VMFD and VMD were comparable in terms of the composition of volatile compounds.

This demonstrates that the use of convection pre-drying to remove large quantities of water from the dried raw materials does not negatively impact the quality of the products dried with the use of the vacuum-microwave technique (Figiel and Michalska 2017). Additionally, the application of microwave-vacuum drying contributes to a significant reduction in drying time compared with the convection method (Calín-Sánchez et al. 2014). Therefore, a combined drying of Pink Rock Rose leaves can be considered to be a favourable compromise that can guarantee high product quality at relatively low production costs in the industrial environment due to the optimally configured line of convection and vacuum-microwaves. However, it should also be noted that the application of the combined method may influence the final chemical composition of the dried herbal product. The use of the CPD-VMFD combination compared with VMD alone resulted in significantly higher concentrations of the main volatile compounds in rosemary (Szumny et al. 2010), dried basil of the highest quality (Calín-Sanchez et al. 2012), and thyme (Calín-Sánchez et al. 2013). In turn, the use of the VMD method only produced the most favourable chemical composition in the dried oregano herb (Figiel et al. 2010) and marjoram (Calín-Sanchez et al. 2015). Accordingly, it seems that the use of convection pre-drying in the preservation of herbs by the vacuum-microwave method significantly contributes to the reduction of process costs, yet the degree of its beneficial effects on the quality of the dried product depends on the chemical composition and the morphological structure of the raw material.

### Colour

The colour of the dried plant materials depends mainly on the presence of natural plant pigments, which are easily degradable during a drying process (Krokida et al. 2001). In our investigations, dried *Cistus creticus* leaves had a slightly lighter colour after the drying process regardless of the method used. However, the dried product generally retained the colour characteristic of the raw material. The colour parameters of the fresh and dried leaves are presented in Table 3.

**Table 3:** Evaluation of the *Cistus creticus* leaves’ color obtained as a result drying of convection (40 °C, 50 °C, 60 °C), vacuum-microwave (VMD 240 W), and vacuum-microwave drying after convection pre-drying (CPD 50 °C-VMFD 240 W)

Drying conditions	L*^1^ ± SD	a*^1^ ± SD	b*^1^ ± SD	∆E ± SD
Fresh	45,99 ± 0.65^a^	− 0.82 ± 0.11^a^	16.14 ± 0.52^a^	Standard
CD 40 °C	52.55 ± 0.93^b^	− 2.86 ± 0.19^b^	13.87 ± 1.03^b*^	7.28 ± 1.07
CD 50 °C	46.94 ± 0.71^a^	− 2.74 ± 0.09^c^	14.82 ± 0.73^c*^	2.63 ± 0.45
CD 60 °C	44.28 ± 0.11^c^	− 1.33 ± 0.09^d^	15.20 ± 0.18^d^	2.02 ± 0.13
VMD 240 W	40.68 ± 0.41^d^	− 0.96 ± 0.04^e^	14.04 ± 0.24^e^	5.72 ± 0.39
CPD 50 °C-VMFD 240 W	45.50 ± 0.55^a^	− 2.12 ± 0v13^f^	15.21 ± 0.24^f^	1.74 ± 0.08

±SD i n = 3

^1^Statistical (*P* < 0.01) differences between the methods, assessed by Statistica 13.1 test Student, are indicated by different letters. *Difference at *P* < 0.05

The type of drying method impacts the ΔL* parameter. We have found that drying using the convection (40 °C, 50 °C, 60 °C) and CPD50 °C-VMFD 240 W methods resulted in a dried product that was not significantly darker than the product obtained by vacuum-microwaves (ΔL* = 40.68). The values of parameter b* indicated that the leaves after drying using all drying methods changed their colour into a light shade of yellow (b* = 13.87–15.21). A slight change in the shade of green (parameter a*) was observed for the leaves dried with all methods. The lowest effect in terms of colour change in comparison with the raw material (the smallest total change in colour) was found in samples obtained using the combined method CPD50 °C-VMFD240 W (a* = 1.74). The largest total colour change occurred when the leaves were dried by convection at 40 °C.

According to other authors, convection and microwave-convection drying produce a colour change in herbs varying from yellow to green (Therdthai and Zhou 2009; Śledź et al. 2013; Sarimeseli 2011). The correlation between the chlorophyll content and the colour parameter a* in dried plant materials was also investigated earlier (Śledź et al.^2013^; Ghumman et al. 2017). A change in the colour of the dried product can result from both enzymatic and non-enzymatic degradation of chlorophyll and carotenoids, and it may include browning which takes place during the drying process (Śledź et al. 2013). Non-enzymatic browning is a consequence of Maillard’s reaction accompanying thermal processes during drying (Wojdyło et al. 2009).

Interestingly, while the increase in the convective drying temperature led to a decrease in the polyphenol content and antioxidant activity as a result of the disintegration of active native plant compounds as determined using the ABTS method, no significant effect on antioxidant activity determined with DPPH was linked to the temperature (Table 2). This result can be attributed to the ability of the free radical scavenging exhibited by certain products of the Maillard reaction (MRPs) which may compensate for the thermal destruction of native phenolic compounds (Wojdyło et al. 2009) as MRPs have already been demonstrated to exhibit antioxidant power by a free radicals-chain breaking type mechanism (Morales and Jimenez-Perez 2001,Yilmaz and Toledo 2005).

## Conclusion

The preservation method of Pink Rock Rose leaves least harmful with respect to the total polyphenol content and the antioxidant activity was convection drying at 40 °C and vacuum-microwave drying at 240 W with the shortest processing time and the lowest specific energy consumption. However, the use of convective pre-drying prior to the vacuum-microwave finish drying led to an increase in the final specific energy consumption and extended the total drying time with an insignificant reduction of the bioactive potential and colour change of the dried final product. We conclude that this combined drying method of Pink Rock Rose leaves may be recommended for implementation in industrial conditions where high-throughput capacity, low specific energy consumption and low overall operating costs are expected.
